# The Cancer Incidence Pattern in Isfahan Province: An Industrial Region in the Central Part of Iran

**DOI:** 10.1155/2024/5592802

**Published:** 2024-11-14

**Authors:** Fereshte Lotfi, Zahra Ravankhah, Hamideh Rashidian, Fotooheh Teimouri, Mohammad Reza Maracy, Shaghyegh Haghjooy Javanmard, Azin Nahvijou, Ali Ghanbari Motlagh, Gholamreza Roshandel, Kazem Zendehdel

**Affiliations:** ^1^Cancer Research Center, Cancer Institute, Tehran University of Medical Sciences, Tehran, Iran; ^2^Department of Medical and Surgical Sciences, University of Bologna, Bologna, Italy; ^3^Population-Based Cancer Registry, Department of Noncommunicable Diseases, Deputy of Health, Isfahan University of Medical Sciences, Isfahan, Iran; ^4^Department of Epidemiology & Biostatistics, School of Public Health, Isfahan University of Medical Sciences, Isfahan, Iran; ^5^Applied Physiology Research Center, Cardiovascular Research Institute, Isfahan University of Medical Sciences, Isfahan, Iran; ^6^Cancer Research Centre, Shahid Beheshti University of Medical Science, Tehran, Iran; ^7^Golestan Research Center of Gastroenterology and Hepatology, Golestan University of Medical Sciences, Gorgan, Iran

## Abstract

**Background:** We aimed to study age-standardized incidence rates (ASRs) of different cancer sites in Isfahan province, an industrialized city in the central part of Iran.

**Method:** We obtained cancer incidence data from 2014 to 2018 in the Isfahan population-based cancer registry (PBCR). We studied quality indicators of PBCR and the validity of residential places for cancer patients. ASRs per 100,000 of common cancers were reported overall and at subprovincial levels.

**Results:** Overall, 42,994 new cancer cases were registered in Isfahan PBCR in 2014–2018, and 51.4% were male. A high percentage of microscopic verification (MV%) (78.1%) and a low percentage of death certificate-only (DCO%) cases (7%) indicate the acceptable validity and completeness of this registry. The ASRs per 100,000 for all cancers combined were 173.7 in men and 171.1 in women. The most common cancers were prostate (ASR = 26.1), bladder (ASR = 19.9), and colorectum (ASR = 19.6) cancers in men and breast (ASR = 49.9), thyroid (ASR = 11.5), and colorectal (ASR = 15.6) cancers in women. Compared to the national reports in Iran, Isfahan province had a higher ASR of leukemia (13.6 in men and 8.9 in women), non-Hodgkin lymphoma (14 in men and 9 in women), and bladder (19.9 in men and 3.4 in women) cancer. The ASR of childhood cancers was 182.7. Notably, the most frequent cancer was leukemia (ASR = 73.4). We observed a wide geographic heterogeneity of cancer incidence in different counties for some cancers like breast, prostate, leukemia, and colorectal cancers.

**Conclusion:** High incidence rates of hematological malignancies and bladder cancers in Isfahan province suggest further research to study the association between occupational and environmental exposures due to industrial pollution.

## 1. Introduction

Cancer remains a significant global health concern, with one in five people worldwide being diagnosed with the disease [1]. Following cardiovascular diseases, cancer is the second cause of death in Iran. Annually, about 130,000 patients are diagnosed with cancer, resulting in 79,000 deaths. The estimated number of cancer patients is projected to rise from 112,000 in 2016 to 160,000 by 2025 in Iran [2]. In addition to established risk factors, including cigarette smoking, obesity, unhealthy diet, aging, and low physical activity, emerging risk factors like opium consumption and waterpipe smoking have been linked to the increased risk of cancer among the Iranian population [3, 4].

The first cancer registry report in Iran dates back to 1956 when the Cancer Institute of Iran was established. In 1969, the first PBCR was established in Iran and reported an incidence rate of higher than 100 per 100,000 person-years for esophageal cancer in the eastern part of the Caspian Littoral region [5, 6]. After an interruption in the early 1980s due to sociopolitical events in Iran, including the revolution and the war with Iraq, a bill was passed by the Iranian Parliament in 1984 mandating reporting of diagnosed or suspected cancers. The Iranian Ministry of Health and Medical Education (MOHME) initiated a pathology-based cancer registry in 1986. Additionally, several attempts were made to establish regional cancer registries in different provinces, including the capital city of Tehran (1998), Ardabil (2000), and Golestan (2000) [6–8]. These efforts revealed a decline in the incidence rates of squamous cell cancer of the esophagus but an increase in the incidence of colon, breast, and distal esophageal cancers [9–12]. Among the others, only the Golestan PBCR resumed operations for an extended period and published the first cancer incidence rates in the Cancer Incidence in Five Continents and provided the opportunity to study long-term trends in the incidence rate of cancers in this population.

In 2013, the MOHME established the integrated Iranian national PBCR, with the first report published in 2018. An updated report on cancer incidence rates between 2013 and 2015, along with predictions until 2025, was subsequently published in 2020 [2]. Significant increases were predicted for thyroid (113.8%), prostate (66.7%), female breast (63.0%), and colorectal (54.1%) cancers in both men and women. In addition, the national PBCR revealed significant disparities in different cancer types across the country. For instance, while breast, colorectal, and prostate cancers were the most common cancers among the population living in the central part of Iran, lung, esophageal, and stomach cancers were higher in the North and Northwest provinces. Furthermore, the incidence rate of stomach cancer in men varied from 39.9 per 100,000 in Ardabil province to 7.18 per 100,000 in Bushehr province and the incidence rate of esophageal cancer varied from14.29 per 100,000 in Golestan province to 1.62 per 100,000 in Charmahal Bakhtiari province. The high-quality estimates of cancer incidence from the national PBCR provided the opportunity to evaluate additional indicators for evidence-based cancer control planning, such as the estimation of population-attributable fraction of modifiable risk factors, and population-based survival in Iran [13, 14].

Given the rich diversity in culture, ethnicity, environmental exposures, lifestyle factors, and socioeconomic status among the Iranian population living in different provinces, analyzing cancer data from each region yields valuable insights into cancer etiology. The aim of this study is to investigate cancer incidence in Isfahan province, a large, industrialized region in central Iran, through an epidemiological perspective. It provides essential baseline data on cancer patterns that can serve as a foundation for future research. The findings may contribute to the development of hypotheses regarding the potential impact of environmental and occupational factors, which are often overlooked in low- and middle-income countries.

## 2. Materials and Methods

### 2.1. Isfahan Province

**Figure 1:**
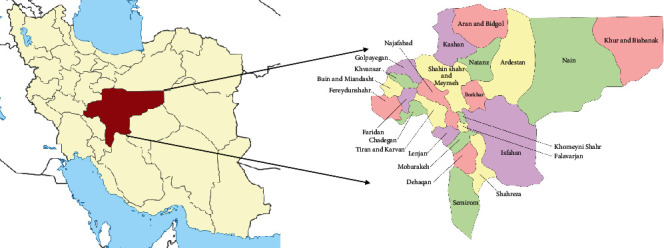
Map of Isfahan province and its 24 counties.

**Figure 2:**
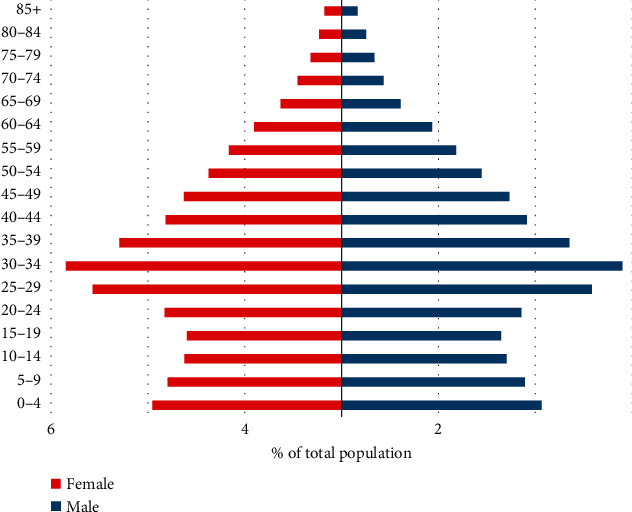
The population distribution pyramid in Isfahan province in 2015–2018 (*Source:* National Population Census).

Isfahan province is the third most populated province in the center of Iran (Figure 1). This province covers an area of approximately 107,018 km^2^ and comprises 24 counties and 106 cities. According to the national census in 2015, the population of Isfahan province was 5,120,850 (Figure 2); approximately 88.00% were urban residents, and 12% were living in rural areas. More than 2.2 million people live in the capital city of Isfahan.

### 2.2. Isfahan Cancer Registry

All Isfahan cancer patients are registered in the population-based cancer registry (PBCR) of Isfahan Province. Isfahan Province initiated a cancer registry in 2005 and published the first report of this program in 2011. The results showed a considerable underestimate of cancer incidence compared to European countries. The Iranian National Population-Based Cancer Registry (INPCR) was launched by the MOHME in the early 2010s. A web-based registration system was used to consolidate the information of all patients in the country, including Isfahan province. Well-trained registry staff in Isfahan PBCR with high expertise in coding and data abstraction collected data from different sources. The International Classification of Diseases for Oncology 3rd edition (ICD-O-3) was used for the classification and coding of cases of cancers recorded. The information on cancer patients in Isfahan PBCR was gathered from three primary sources, including pathology and cytology reports, hospital files from 35 hospitals (private or educational), radiotherapy and imaging centers, and death certificate reports. In this analysis, we used data on cancer patients registered in Isfahan PBCR from 2014 to 2018 (Figure S1). However, after careful evaluation of the data quality, we found that data in the first year (2014) was incomplete and was excluded from this study.

### 2.3. Quality Control

As part of the national program, Isfahan PBCR adhered to the International Agency for Research on Cancer (IARC) guidelines for coding and quality control processes [15]. We used the percentages of microscopic verification (MV%) and death certificate-only cases (DCOs%) to evaluate the quality of Isfahan PBCR. We compared the age-specific curve with an expected range using standard age-specific curves of CI5 Volume 8 overall and for common cancers. We studied the mean notification per case/number of sources. Additionally, we examined the stability of incidence rates over 5 years. To study the accuracy of residential addresses registered for patients, we randomly selected 10% of patients registered in the Isfahan PBCR through simple random sampling and telephone interviews with the patients or their next of kin to evaluate the addresses recorded for each patient.

### 2.4. Statistical Analysis

We estimated age-specific and age-standardized incidence rates (ASRs) per 100000. The age-specific incidence rate was calculated for five age categories (< 30 years, 30–44 years, 45–59 years, 60–74 years, and ≥ 75 years). We used weights of the standard world population in 2000 in 5-year groups by gender to estimate the ASR. We studied the ASR for common cancers in Isfahan province overall and in different cities. We used CanReg5 Ver. 5.00.44h software, developed by the IARC in Lyon, France, for statistical analyses.

## 3. Result

The average annual population in Isfahan province between 2015 and 2018 was 5,120,850 (2,599,477 males and 2,521,373 females). Overall, 42,994 cases of malignant tumors were registered in Isfahan PBCR in 2015–2018, of which 51.4% were men and 48.5% were women. The overall percentage of DCO was 7.6%, and MV% was 76%. The DCO-flagged proportion varied from 32.9% in liver cancer to 0% in testis cancer. The highest MV% was observed in corpus uteri (92.5%) and breast (92.2%), and the lowest MV% was observed in liver cancer (29.5%) (Table S1).

**Figure 3:**
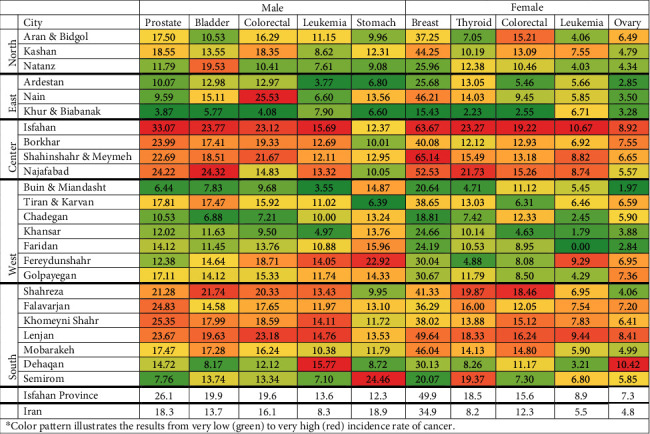
Age-standardized incidence rates (ASRs) of the most common cancers in different counties of Isfahan province in 2015–2018⁣^∗^.

The ASR for all-cancer excluding nonmelanoma skin cancer (NMSC) were 173.7 and 171.1 per 100,000 in men and women, respectively. In women, breast cancer was the most common cancer (*N* = 5627, ASR = 49.9), followed by thyroid (*N* = 2252, ASR = 18.5) and colorectal (*N* = 1699, ASR = 15.6) cancers. Among men, prostate cancer was the leading cancer site (*N* = 2,804, ASR = 26.1), followed by bladder (*N* = 2152, ASR = 19.9) and colorectal (*N* = 2185, ASR = 19.9) cancers. The ASRs for other common cancers in men, including prostate, bladder, and colorectal cancers, were higher in Isfahan province than the overall rates reported for the Iranian population (Figure 3). Almost all cancer sites in Isfahani women, except for stomach and lung cancers, were higher than the average incidence rate in Iranian women.

Estimation of ASR for different cities in Isfahan province showed that the ASR of most cancer sites ranked first in the capital city of Isfahan for men and women (Figure 3) (Tables S2 and S3). ASR of breast cancer was higher than 60 per 100,000 in Isfahan (ASR = 65.1) and Shahin Shahr (ASR = 63.7 per 100,000) cities, while it was lower than (ASR = 15.4) in Khur and Biabanak cities. Also, the highest ASR of thyroid cancer, the second most common malignancy in Isfahan, was observed in Isfahan (ASR = 23.3) and Najaf Abad (ASR =21.7) cities. The highest ASRs among men in Isfahan province were prostate, bladder, and colorectal cancers, which mainly belong to Isfahan (ASR = 33.07 per 100,000), Najaf Abad (ASR = 24.3), and Nain (ASR = 25.5) cities. Isfahan city had the highest incidence rate of leukemia in men (ASR = 15.7 per 100,000) and women (ASR = 10.7 per 100,000). In addition, the incidence rate of leukemia was high among men living in Dehaqan (ASR = 15.8), Lenjan (ASR = 14.8), Komeinishahr (ASR = 14.1), and Fereidoonshahr (ASR = 14.1). It was also high among women living in Lenjan (ASR = 9.4) and Fereidoonshahr (ASR = 9.2). The incidence rate of non-Hodgkin lymphoma (NHL) was also high in Isfahan city, both in men (ASR = 8.3) and women (ASR = 5.5). In addition, the ASR for NHL was high in men of Flavarjan (ASR = 7.7), Feridan (ASR = 7.3), women of Nain (ASR = 7.3), and Shahinshar. We observed a high incidence rate of stomach cancer among men of Semirom (ASR = 24.5), an agricultural region located in the southern part of Isfahan. The incidence rates for all cancer sites, categorized by age group and sex, are presented in Tables 1 and 2.

Table 3 presents the incidence of childhood cancer in the Isfahan province. Incidence rates were 1.3-fold higher in boys than in girls. The ASR of childhood cancers was 182.7 per 1000000. Notably, the most frequent cancer was leukemia (ASR = 73.4 per 1,000,000), followed by lymphomas (ASR = 25.7 per 1,000,000), and CNS cancers (ASR = 24.9 per 1,000,000).

## 4. Discussion

We investigated cancer incidence rates in Isfahan province, a central industrial region in Iran. Our findings diverged from the national trends. Among men, prostate, colorectal, bladder, lymphoma, and stomach cancers had the highest ASRs, while breast, thyroid, colorectal, lymphoma, and leukemia were more common among women. Notably, Isfahan's industrialized city exhibited elevated rates of hematological malignancies. Additionally, leukemia, lymphoma, and CNS cancers were prevalent among Isfahani children. Our study also highlighted geographic disparities in cancer incidence rates within the province.

We found that Isfahan PBCR has high-quality data. The proportion of cancer cases identified with DCO% was considerably low and the proportion of MV% was very high, indicating high validity and completeness of the Isfahan PBCR [16, 17]. Despite the low quality of PBCRs in the EMR region, some countries in the region showed a high MV, including Kuwait (90%), Jordan (96%), Turkey (81%), Qatar (88%), Bahrain (88%), and Saudi Arabia (91%), Israel (90%) are more reliable owing to the higher proportion of morphological verification. Altogether, at the national level, we still lag behind global standards in the quality of registered cancer cases [18, 19].

A high ASR of breast, prostate, and colorectal cancers, which accounts for approximately one-third of the Isfahan cancer incidence rate, is correlated with diagnostic activities linked to the high human development index (HDI) in this population compared to other provinces in Iran [20]. In the 2021 human development report, Iran is classified as a high HDI country. However, the distribution of HDI status across provinces was highly varied, ranging from 0.66 in Sistan-Baluchestan province to 0.81 in the capital city of Tehran in 2020 [21]. Isfahan province's HDI was 0.80, ranked second after the capital city of Tehran [22]. Improvements in socioeconomic status and the HDI are associated with higher cancer incidence rates overall, particularly for hormone-related cancers such as prostate, colorectal, and breast cancer. Additionally, enhanced access to diagnostic facilities in a developed city like Isfahan would improve the detection and registration of these cancers.

While female breast cancer remains the most frequently diagnosed cancer globally, the incidence rates across Western Asia and the Eastern Mediterranean Region (EMR) exhibit a similar pattern, with an ASR of about 45 per 100,000, which is almost half of those in developed countries in North America and Europe [23]. Urbanization and its negative consequences, including changing dietary patterns to consuming excess fat and sugar and increasing sedentary lifestyles, have a prominent role in increasing the risk of breast cancer in high HDI countries [24]. ASR of breast cancer was almost 40% higher in Isfahan province than in the average rate reported for the Iranian population [2]. It seems this ratio reflects a combination of demographic factors allied to economic and social development and also awareness and early detection in this province. Addressing the completeness of the PBCRs and the proportion of patients with early-stage breast cancer in different provinces will help to better understand this disparity.

Although stomach cancer was the most common malignancy among Iranian men in 2016, a prediction study revealed that prostate cancer would take the lead in 2025 [2]. Prostate cancer is the most common cancer in males living in Isfahan and some other more developed provinces in Iran. An examination of the global profile of prostate cancer across 20 regions reveals that the incidence rates are notably lower in all four subgroups of Asian countries including Eastern Asia, South-Eastern Asia, South-Central Asia, and Western Asia. Conversely, European countries exhibit incidence rates almost 3-fold higher (ASR 80) than the Western Asia (ASR 28.6) [1]. Prostate cancer is diagnosed in more advanced stages in low- and middle-income countries (LMICs) than the high-income countries, leading to a poorer prognosis in the LMICs [25]. A study of cancer survival in Iran showed that the 5-year survival rate of prostate cancer was 74.9%, which is lower than the rate reported by the United States (97.7%), Japan (93.0%), and Finland (93.2%) [13, 15, 18]. Considering the inconsistent evidence in the major screening trials regarding the decreasing mortality from prostate cancer [26], until the introduction of an appropriate screening test, it is essential to implement public awareness, timely diagnosis, and effective treatment programs to decrease the mortality of prostate cancer.

Studies have found remarkable differences in the distribution of gastrointestinal cancers in Iran. North and Northwest Iran have the highest incidence rate of stomach cancers; however, colorectal cancer is the first gastrointestinal cancer in the central part of Iran [15]. Beyond the significant genetic basis, increasing a wide range of Western lifestyle-related risk factors such as sedentary behaviors, fiber-deficient diet, obesity, and consumption of red or processed meat as known as risk factors for colorectal cancer accounts for the growing incidence of this cancer over the next decade in Iran [2, 27, 28]. Colorectal cancer is the second most common cancer in men and the third most frequent cancer in women of Isfahan province. Still, there is regional diversity at the subprovincial level, as the more industrialized cities located in the Southern and Western parts of Isfahan province, showed the highest incidence rate of colorectal cancers. The incidence rate of colorectal cancer, the most commonly diagnosed cancer among both men and women in this province, aligns with the patterns observed in other high HDI countries in Western Asia. Nevertheless, it is worth noting that it is still half the rates reported in North America, Europe, and Oceania [23]. As observed in Golestan PBCR and predicted at the national level, the incidence rate will likely increase rapidly in Iran and require prompt action to prevent and control colorectal cancer in Isfahan province [2, 29].

The incidence rate of thyroid cancer was higher among women living in Isfahan province. The risk of “overdiagnosis” of thyroid cancer has long been controversial worldwide. In South Korea, this proportion was estimated to be up to 90% [30]. We also found a higher incidence rate of thyroid cancer in Isfahani women aged 45–59, indicating the role of overdiagnosis among women living in Isfahan province. Another indication of the role of overdiagnosis is that thyroid cancer is higher in Isfahan and Najafabad cities, where the access to a specialist is higher, and women may receive a higher diagnostic measure in these areas. However, the high ASR of thyroid cancer in Isfahan could be linked to environmental exposures. We suggest designing studies to investigate the reason for the high incidence rate of thyroid cancer in Isfahan province and estimate the role of risk factors and overdiagnosis on the burden of thyroid cancer in Isfahan and other high-incidence regions.

Isfahan is an industrial metropolis in Iran. Hence, there is a high probability of occupational and environmental exposure among residents of this province. These exposures are mostly associated with an increased risk of leukemia, lymphoma, and bladder cancers, all of which have a substantially high incidence rate in this province. High industrial activities resulting in ambient air pollution in these areas can potentially increase the risk of leukemia cancer [31, 32]. Notably, over 90% of countries in the EMR reported leukemia cancer incidence rates of less than 10 per 100,000. This observation underscores that the incidence rate of this cancer in Isfahan exceeds the regional average [23]. The incidence rate of stomach cancer is very high in the central and southern parts of Isfahan province, where agricultural activities are common. This finding supports the previous report that stomach cancer was very high in the northern and northwestern parts of Iran [33, 34]. While *Helicobacter pylori* infection is the main risk factor for stomach cancer, exposure to pesticides and the nitrate compound available in fertilizers among agricultural workers may play a role in this observation [35–37].

A higher ASR of bladder cancer in Isfahani men than in the average rate reported for Iran can be linked to the industrial nature of the province and exposure to occupational risk factors, including polycyclic aromatic hydrocarbons, aromatic amines, and chlorinated hydrocarbons in the numerous rubber and petroleum industries in the center and south of the province [38]. Tobacco smoking is the strongest risk factor for bladder cancer, and the geographic distribution map of the bladder cancer incidence rate in Iran also substantiates this association [2, 39]. In addition, to cigarettes, waterpipe smoking plays an important role in the development of bladder cancer in the Eastern Mediterranean region, including Iran [14, 40]. The highest ASR of bladder cancer belongs to the provinces with a high prevalence of tobacco use, like Kerman, Qom, Markazi, Bushehr, and Fars. Also, it seems the proximity of the center and south of Isfahan, including Isfahan, Najafabad, Shahreza, and Lenjan cities to Kerman province, wherein the prevalence of opium is high, can explain these findings. Opium use was reported to be a strong risk factor for several cancers, including bladder, lung, and laryngeal cancer [41]. However, since the lung cancer incidence rate was generally low in Isfahan province, the excess risk attributed to occupational and environmental exposures from industries presents a more plausible explanation.

Leukemia, lymphoma, and CNS neoplasms rank among the most prevalent childhood cancers globally. Our study identified a significantly higher incidence of leukemia in Isfahani children compared to children in other countries. The incidence rate of childhood leukemia in Isfahan is similar to the highest rates observed globally, such as Hispanic white children in the USA [2, 42]. This observation warrants conducting epidemiological and clinical research to understand the reason behind this higher burden of leukemia. In addition, it is important to conduct clinical research, enhance the quality of care for this cancer, and improve patient outcomes.

The strengths of this study include using a high-quality cancer registry to study the geographical pattern of cancer in a large industrial province. For the first time, we studied the variation of cancer incidence rates at the city level in Iran and demonstrated the importance of this type of analysis. However, the Isfahan PBCR is still young, and the sample size at the city level for different cancers is still low, precluding the assessment of temporal trends in this analysis. In addition, breaking the data to the city level led to a small number of cancers in small cities. Consequently, caution is advised when interpreting results derived from the smaller cities. Replication of this analysis will be necessary as more data becomes available in the coming years. However, given the quality of data in this region, we suggest conducting cohort and case-control studies based on the hypothesis generated in this study to uncover the etiology of different cancer types in Isfahan province.

In conclusion, to the best of our knowledge, this is the first study that reports variation in cancer incidence across cities in Isfahan province, an industrialized province in the center of Iran. The high incidence rate of cancers in Isfahan province, particularly its status as a hotspot for hematological malignancies in both adults and children, demands special attention. Further epidemiological research is necessary to understand the underlying reasons behind these findings. Additionally, public authorities should intervene to prevent these cancers and enhance patient outcomes.

## Figures and Tables

**Table 1:** Incidence rates for all cancer sites by age groups among men in Isfahan province in 2015–2018.

**Cancer site**	**ICD (10th)**	**All ages**		**Age-specific incidence rates**				
		**Number of patients**	**ASR per 100,000**	**< 30**	**30–44**	**45–59**	**60–74**	**≥ 75**
Lip, oral cavity, and pharynx	C00–C10, C12–C14	311	2.5	1.7	3.6	14.3	37	102.3
Nasopharynx	C11	66	0.6	0.9	0.9	4.8	5.5	8.2
Oesophagus	C15	313	2.8	0.1	1.2	10.2	54.5	124.4
Stomach	C16	1405	12.3	0.9	10.9	61	201.6	506.5
Colorectal	C18-C21	2185	19.9	3	20.3	106.1	365.4	515.2
Liver	C22	433	3.9	1.6	2.5	17.4	64.4	157.5
Pancreas	C25	478	4.4	0.6	2.1	19.2	85.5	148.4
Larynx	C32	357	3.3	0.3	2.1	24.5	54	66.3
Trachea, bronchus, and lung	C33–34	1221	11.1	1.3	5.6	57.2	203.2	376.7
Bone	C40–41	154	1.5	6.7	3.1	4.4	10.2	29.5
Melanoma of skin	C43	106	0.9	1	1.1	4.5	12.4	31.7
Nonmelanoma skin	C44	3061	28.2	2.9	19.7	108.2	567.9	942.4
Prostate	C61	2804	26.1	0.4	2	63.7	599.3	1123.7
Testis	C62	270	2.1	11.3	12.1	6.8	4.2	2.2
Kidney	C64–C66, C68	513	4.9	2.9	6.1	28.7	79.2	86.2
Bladder	C67	2152	19.9	2.3	11	109.2	373.2	534.6
Brain, nervous system	C70-72	845	7.7	17.2	19.2	38.4	81.1	117.1
Thyroid	C73	498	4.2	9.9	18	24	33.7	22.6
Lymphoma	C81–C85,C88,C90,C96	1526	14	31.9	23.2	69.7	175.5	239.7
Leukemia	C91–C95	1435	13.6	36.4	18.2	54.7	154.6	324.7
All sites	ALL	22112	201.9	150.1	205.7	905.7	3432.4	6024.1
All sites but C44	ALLbC44	19051	173.7	147.2	186.1	797.5	2864.5	5081.8

**Table 2:** Incidence rates for all cancer sites by age groups among women in Isfahan province in 2015–2018.

**Cancer site**	**ICD (10th)**	**All ages**		**Age-specific incidence rates**				
		**Number of patients**	**ASR per 100,000**	**< 30**	**30**–**44**	**45**–**59**	**60**–**74**	**≥ 75**
Lip, oral cavity, and pharynx	C00–C10, C12–C14	275	2.5	1.7	5.1	9.5	38.8	74.3
Nasopharynx	C11	24	0.2	0.5	0.4	1.8	1.3	3
Esophagus	C15	179	1.6	0.2	1.2	6.7	26.7	73.8
Stomach	C16	679	5.9	2.2	8.5	31.3	82.8	237
Colorectal	C18–C21	1699	15.5	3.1	24.3	97.4	243.9	348.7
Liver	C22	341	3	1.5	2.9	12.2	41	154
Pancreas	C25	368	3.3	0.5	3.7	14.5	57.2	125.1
Larynx	C32	42	0.4	0.1	0.1	1.6	4.5	18.6
Trachea, bronchus, and lung	C33–34	560	4.9	1.7	4.6	19.2	76.7	240.5
Bone	C40–41	130	1.3	6.3	2.5	4.1	10	19.1
Melanoma of skin	C43	106	1	0.5	1	4.6	17.1	27.1
Nonmelanoma skin	C44	1840	17.3	2.6	14.1	71.2	357.4	472
Breast	C50	5627	49.9	10.8	184.2	413.7	497.3	350.2
Cervix uteri	C53	209	1.8	0.9	7.6	14.3	15.5	18.8
Uterus	C54-C55	839	8	0.8	11.4	65.6	120.5	69.1
Ovary	C56	806	7.3	7.8	17.8	56	78.8	69.2
Kidney	C64-C66, C68	325	3.1	2.3	5.3	18.9	50	38.5
Bladder	C67	388	3.4	1.2	3.5	16.2	57.3	130.4
Brain, nervous system	C70–72	692	6.4	14.3	15.6	27.7	79.3	110
Thyroid	C73	2252	18.5	50.8	108	103.8	77.2	44.6
Lymphoma	C81–C85, C88, C90, C96	976	9	18.7	18.2	46.1	113.7	123.9
Leukemia	C91–C95	887	8.9	30.5	14.1	28.2	92.5	166.6
All sites	ALL	20882	188.4	179.8	477.8	1132.8	2336	3331
All sites but C44	ALLbC44	19042	171.1	177.3	463.6	1061.5	1978.7	2859.2

**Table 3:** Incidence of childhood cancer, classified according to the International Classification of Childhood Cancer (ICCC-3).

**ICCC3**		**Number of cases**						**Incidence rate per million**				
**Group**	**Cancer site**	**Age group (year)**			**Overall**			**Age group (year)**			**Overall**	
		**0–4**	**5–9**	**10–14**	**Number**	**M/F**	**% total**	**0–4**	**5–9**	**10–14**	**Crude**	**ASR**
I	Leukemia	155	115	50	320	1.2	40.0	96.4	78.0	37.6	72.5	73.4
II	Lymphoma	27	43	45	115	2.5	14.4	16.8	29.2	33.8	26.1	25.7
III	CNS neoplasms	40	43	27	110	1.4	13.7	24.9	29.2	20.3	24.9	24.9
IV	Neuroblastoma	20	9	0	29	0.7	3.6	12.4	6.1	0.0	6.6	6.8
IX	Soft tissue sarcomas	12	14	7	33	0.8	4.1	7.5	9.5	5.3	7.5	7.5
V	Retinoblastoma	14	0	0	14	1.0	1.7	8.7	0.0	0.0	3.2	3.4
VI	Renal tumors	24	8	0	32	1.7	4.0	14.9	5.4	0.0	7.2	7.5
VII	Hepatic tumors	7	4	1	12	1.4	1.5	4.4	2.7	0.8	2.7	2.8
VIII	Malignant bone tumors	2	7	25	34	0.9	4.2	1.2	4.7	18.8	7.7	7.5
X	Germ cell tumors	11	4	11	26	0.9	3.2	6.8	2.7	8.3	5.9	5.9
XI–XII	Other	35	18	22	75	1.0	9.4	21.8	12.2	16.5	17.0	17.2
	Unknown	1	0	0	1	0.0	0.1	0.6	0.0	0.0	0.2	0.2
	All sites	348	265	188	801	1.3	100.0	216.5	179.7	141.2	181.5	182.7

## Data Availability

The data that support the findings of this study are available from the first author (F.L.) upon reasonable request.

## Supporting Information

Supporting Information Additional supporting information can be found online in the Supporting Information section. Figure S1: result of the number of all cancers (excluding nonmelanoma skin cancer) by calendar year in Isfahan Cancer Registry. Table S1: result of the indices of data quality: percentages of cases with microscopic verification (MV), clinical and death certificate only (DCO) in the Isfahan population-based cancer registry (2015–2018). Table S2: result of the population size, total number of patients, and age-standardized incidence rates (ASR) of the most common cancers in different counties of Isfahan province among men in 2015–2018. Table S3: result of the population size, total number of patients, and age-standardized incidence rates (ASR) of the most common cancers in different counties of Isfahan province among women in 2015–2018.
