# Supplementary material for: The Cancer Incidence Pattern in Isfahan Province: An Industrial Region in the Central Part of Iran
Source: J Cancer Epidemiol. 2024 Nov 14;2024:5592802. doi: 10.1155/2024/5592802 (PMC11581794; doi:10.1155/2024/5592802)
Supplement: Supporting Information — Additional supporting information can be found online in the Supporting Information section. Figure S1: result of the number of all cancers (excluding nonmelanoma skin cancer) by calendar year in Isfahan Cancer Registry. Table S1: result of the indices of data quality: percentages of cases with microscopic verification (MV), clinical and death certificate only (DCO) in the Isfahan population-based cancer registry (2015–2018). Table S2: result of the population size, total number of patients, and age-standardized incidence rates (ASR) of the most common cancers in different counties of Isfahan province among men in 2015–2018. Table S3: result of the population size, total number of patients, and age-standardized incidence rates (ASR) of the most common cancers in different counties of Isfahan province among women in 2015–2018. [file 5592802.f1.docx]

**Figure 1- Number of all cancers (excluding non-melanoma skin cancer) by calendar year in Isfahan Cancer Registry**

**Table 1- Indices of data quality: percentages of cases with microscopic verification (MV), clinical and death certificate only (DCO) in Isfahan population-based cancer registry (2015-2018)**

| Cancer site | ICD-10 | No. Cases | % Total | Basis of diagnosis | | |
| --- | --- | --- | --- | --- | --- | --- |
|  |  |  |  | % DCO | % Clinical | % M.V |
| Mouth & pharynx | C00-14 | 676 | 2.0 | 4.4 | 11.5 | 84.0 |
| Oesophagus | C15 | 492 | 1.4 | 8.9 | 14.8 | 76.2 |
| Stomach | C16 | 2,084 | 6.1 | 12.5 | 13.8 | 73.7 |
| Colon, rectum, anus | C18-21 | 3,884 | 11.3 | 5.2 | 11.9 | 83.0 |
| Liver | C22 | 774 | 2.3 | 32.9 | 37.6 | 29.5 |
| Pancreas | C25 | 846 | 2.5 | 16.3 | 40.4 | 43.3 |
| Larynx | C32 | 399 | 1.2 | 13.0 | 12.3 | 74.7 |
| Trachea, bronchus, lung | C33-34 | 1,781 | 5.2 | 23.9 | 29.1 | 47.0 |
| Melanoma of skin | C43 | 212 | 0.6 | 0.5 | 1.4 | 98.1 |
| Breast | C50 | 5,708 | 16.6 | 2.2 | 5.6 | 92.2 |
| Cervix | C53 | 209 | 0.6 | 3.8 | 8.6 | 87.6 |
| Corpus & uterus NOS | C54-55 | 839 | 2.4 | 2.7 | 4.8 | 92.5 |
| Ovary & adnexa | C56 | 806 | 2.4 | 2.7 | 22.1 | 75.2 |
| Prostate | C61 | 2,804 | 8.2 | 11.2 | 11.7 | 77.1 |
| Testis | C62 | 270 | 0.8 | 0.0 | 16.3 | 83.7 |
| Kidney & urinary NOS | C64-66,68 | 848 | 2.5 | 4.5 | 13.8 | 81.7 |
| Bladder | C67 | 2,540 | 7.4 | 2.0 | 11.2 | 86.8 |
| Brain & central nervous system | C70-72 | 1,537 | 4.5 | 21.7 | 36.8 | 41.6 |
| Thyroid | C73 | 2,750 | 8.0 | 0.4 | 6.2 | 93.4 |
| Lymphoma | C81-85,90,88,96 | 2,502 | 7.3 | 2.5 | 22.9 | 74.6 |
| Leukaemia | C91-95 | 2,322 | 6.8 | 7.5 | 9.3 | 83.2 |
| All sites | All | 34,283 | 100.0 | 7.5 | 14.4 | 78.1 |

**Table 2-Population size, total number of patients, and age-standardized incidence rates (ASR) of the most common cancers in different counties of Isfahan province among men in 2015-2018 ^*^**

|  | City | Population | All cases | NHL | Lung | Brain | Kidney | Esophagus |
| --- | --- | --- | --- | --- | --- | --- | --- | --- |
| North | Aran & Bidgol | 52644 | 269 | 6.56 | 5.68 | 5.58 | 0.27 | 3.21 |
|  | Kashan‎ | 184806 | 1206 | 2.97 | 8.30 | 9.36 | 2.36 | 3.83 |
|  | Natanz | 22322 | 164 | 5.84 | 7.90 | 7.86 | 0.98 | 5.55 |
| East | Ardestan | 21281 | 156 | 2.98 | 11.24 | 8.71 | 2.63 | 1.29 |
|  | Nain | 19618 | 168 | 5.63 | 16.91 | 9.86 | 0.00 | 3.88 |
|  | Khur & Biabanak | 9977 | 49 | 5.68 | 5.08 | 3.09 | 2.48 | 4.28 |
| Center | Isfahan‎ | 1133446 | 11376 | 8.30 | 11.22 | 7.03 | 5.53 | 2.11 |
|  | Borkhar | 63109 | 439 | 7.47 | 13.69 | 5.80 | 2.70 | 5.66 |
|  | Shahinshahr & Meymeh | 117467 | 954 | 4.17 | 11.96 | 5.92 | 5.01 | 2.49 |
|  | Najafabad‎ | 161791 | 1217 | 6.34 | 11.50 | 9.65 | 2.85 | 2.96 |
| West | Buin & Miandasht | 12291 | 74 | 3.46 | 5.25 | 3.55 | 3.52 | 0.75 |
|  | Tiran & Karvan | 36701 | 224 | 1.61 | 13.14 | 3.69 | 1.23 | 2.14 |
|  | Chadegan | 16554 | 79 | 1.42 | 6.14 | 7.72 | 4.19 | 1.80 |
|  | Khansar | 16446 | 140 | 0.00 | 13.57 | 6.52 | 1.44 | 2.01 |
|  | Faridan | 25685 | 198 | 7.29 | 15.30 | 11.05 | 5.14 | 2.66 |
|  | Fereydunshahr | 18171 | 138 | 2.65 | 4.20 | 9.64 | 1.56 | 4.58 |
|  | Golpayegan | 45913 | 370 | 2.29 | 9.49 | 4.73 | 0.73 | 3.17 |
| South | Shahreza | 81640 | 653 | 5.09 | 12.65 | 5.37 | 4.10 | 4.31 |
|  | Falavarjan | 128785 | 931 | 7.72 | 13.71 | 6.56 | 2.95 | 3.76 |
|  | Khomeyni Shahr | 164787 | 1146 | 4.86 | 11.01 | 6.34 | 5.04 | 4.73 |
|  | Lenjan | 133557 | 1039 | 6.74 | 12.60 | 7.99 | 5.42 | 2.24 |
|  | Mobarakeh | 77029 | 546 | 5.01 | 15.05 | 7.25 | 5.51 | 2.41 |
|  | Dehaqan | 17571 | 109 | 3.84 | 9.22 | 3.46 | 3.73 | 1.65 |
|  | Semirom | 37886 | 244 | 4.08 | 6.57 | 4.95 | 6.60 | 2.03 |
|  | Isfahan Province | 2599477 | 22112 | 7.1 | 11.1 | 7.7 | - | - |
|  | Iran^**^ | 40498442 | 62592 | 5.3 | 10.7 | 6.5 | - | - |
| *Color pattern illustrates the results from very low (green) to very high (red) incidence rate of cancer.  ** Cancer in Iran 2008 to 2025: Recent incidence trends and short-term predictions of the future burden. PMID:33884608 | | | | | | | | |

**Table 3-Population size, total number of patients, and age-standardized incidence rates (ASR) of the most common cancers in different counties of Isfahan province among women in 2015-2018^*^**

|  | City | Population | All cases | NHL | Corpus | Brain | Stomach | Lung |
| --- | --- | --- | --- | --- | --- | --- | --- | --- |
| North | Aran & Bidgol | 50873 | 261 | 0.94 | 7.59 | 7.62 | 3.41 | 2.78 |
|  | Kashan‎ | 179676 | 1189 | 1.87 | 9.76 | 8.15 | 6.28 | 2.97 |
|  | Natanz | 21655 | 131 | 2.87 | 6.89 | 6.23 | 5.39 | 2.04 |
| East | Ardestan | 20824 | 129 | 3.43 | 6.76 | 3.51 | 6.40 | 1.12 |
|  | Nain | 19643 | 183 | 7.25 | 3.40 | 10.26 | 10.15 | 7.62 |
|  | Khur & Biabanak | 9784 | 46 | 0.00 | 2.61 | 2.61 | 5.21 | 5.39 |
| Center | Isfahan‎ | 1109803 | 11251 | 5.49 | 9.73 | 5.22 | 6.12 | 5.95 |
|  | Borkhar | 59310 | 352 | 3.65 | 4.53 | 5.42 | 2.79 | 3.97 |
|  | Shahinshahr & Meymeh | 117200 | 1014 | 4.40 | 7.65 | 5.41 | 5.82 | 3.37 |
|  | Najafabad‎ | 157414 | 1223 | 2.17 | 9.39 | 7.46 | 4.36 | 4.13 |
| West | Buin & Miandasht | 11872 | 54 | 1.50 | 1.50 | 8.34 | 3.54 | 2.51 |
|  | Tiran & Karvan | 34874 | 222 | 2.33 | 6.71 | 5.07 | 6.73 | 2.42 |
|  | Chadegan | 15925 | 73 | 1.67 | 2.84 | 1.28 | 9.72 | 2.81 |
|  | Khansar | 16603 | 111 | 2.84 | 2.48 | 4.93 | 7.53 | 7.00 |
|  | Faridan | 24205 | 149 | 2.58 | 2.76 | 5.26 | 8.59 | 7.66 |
|  | Fereydunshahr | 17483 | 106 | 3.02 | 5.03 | 4.38 | 8.21 | 8.54 |
|  | Golpayegan | 44173 | 325 | 1.83 | 5.74 | 8.68 | 7.92 | 4.26 |
| South | Shahreza | 78157 | 649 | 4.06 | 7.11 | 7.21 | 4.09 | 4.50 |
|  | Falavarjan | 121029 | 797 | 3.28 | 3.73 | 4.75 | 5.35 | 6.31 |
|  | Khomeyni Shahr | 154940 | 969 | 2.67 | 5.37 | 3.23 | 6.75 | 4.54 |
|  | Lenjan | 129355 | 986 | 3.73 | 6.63 | 5.53 | 6.11 | 4.70 |
|  | Mobarakeh | 73412 | 522 | 2.65 | 6.59 | 7.12 | 4.73 | 3.67 |
|  | Dehaqan | 16940 | 108 | 2.61 | 5.39 | 10.93 | 4.62 | 0.66 |
|  | Semirom | 36223 | 215 | 2.23 | 2.29 | 4.33 | 5.59 | 4.17 |
|  | Isfahan Province | 2521373 | 20882 | 4.2 | - | 6.4 | 5.9 | 4.9 |
|  | Iran^**^ | 39427828 | 60102 | 3.3 | - | 4.9 | 8.8 | 5.0 |
| *Color pattern illustrates the results from very low (green) to very high (red) incidence rate of cancer.  ** Cancer in Iran 2008 to 2025: Recent incidence trends and short-term predictions of the future burden. PMID:33884608 | | | | | | | | |
